# Nonprimate Hepaciviruses in Domestic Horses, United Kingdom

**DOI:** 10.3201/eid1812.120498

**Published:** 2012-12

**Authors:** Sinéad Lyons, Amit Kapoor, Colin Sharp, Bradley S. Schneider, Nathan D. Wolfe, Geoff Culshaw, Brendan Corcoran, Bruce C. McGorum, Peter Simmonds

**Affiliations:** Author affiliations: University of Edinburgh, Edinburgh, Scotland, UK (S. Lyons, C. Sharp, G. Culshaw, B. Corcoran, B.C. McGorum, P. Simmonds);; Columbia University, New York, New York, USA (A. Kapoor);; Global Viral Forecasting Initiative, San Francisco, California, USA (B.S. Schneider, N.D. Wolfe)

**Keywords:** hepatitis C virus, viruses, Equus ferus caballus, Canis familiaris, canine, hepacivirus, CHV, zoonosis, evolution, primate, viral hepatitis, horse, dog, United Kingdom, non-primate, nonprimate, hepacivirus, NPHV, gb virus b, gbv-b

## Abstract

Although the origin of hepatitis C virus infections in humans remains undetermined, a close homolog of this virus, termed canine hepacivirus (CHV) and found in respiratory secretions of dogs, provides evidence for a wider distribution of hepaciviruses in mammals. We determined frequencies of active infection among dogs and other mammals in the United Kingdom. Samples from dogs (46 respiratory, 99 plasma, 45 autopsy samples) were CHV negative by PCR. Screening of 362 samples from cats, horses, donkeys, rodents, and pigs identified 3 (2%) positive samples from 142 horses. These samples were genetically divergent from CHV and nonprimate hepaciviruses that horses were infected with during 2012 in New York state, USA. Investigation of infected horses demonstrated nonprimate hepacivirus persistence, high viral loads in plasma (10^5^–10^7^ RNA copies/mL), and liver function test results usually within reference ranges, although several values ranged from high normal to mildly elevated. Disease associations and host range of nonprimate hepaciviruses warrant further investigation.

## Abstract 2

Viruses related to human hepatitis C virus infect horses in the United Kingdom without evidence of hepatic or other systemic disease.

Hepatitis C virus (HCV) is a positive-sense RNA virus, classified as the type member of the family *Flaviviridae* and the genus *Hepacivirus*. HCV is a major human pathogen, causing persistent infections that target and eventually destroy the liver in a substantial proportion of those infected (1,2). HCV infections are distributed worldwide and have spread epidemically within the past 40–60 years within western countries through blood-borne routes such as blood and blood product transfusion and injection drug use.

HCV shows considerable genetic diversity; 7 genotypes show >30% nt sequence divergence from each other (3). Several of these genotypes are associated with suspected endemic source areas in central and western sub-Saharan Africa (genotypes 1, 2, and 4) (4–7) and Southeast Asia (genotypes 3 and 6) (8,9). These regions harbor the greatest diversity of HCV subtypes, implying a long-term, endemic circulation of the virus for several hundred years. The spread of certain genotype variants from these populations, such as 1a and 1b to Western countries, 3a among injection drug users in Europe, and 4a to Egypt, where it was extensively transmitted by medical injections (10–14), show several parallels with the emergence and rapid spread of HIV-1 among new risk groups from a central African reservoir over a similar time frame (15).

Based on this model, it has been frequently speculated that HCV could have an ultimate animal origin in >1 nonhuman primate species, in much the same way as human HIV-1 originated from chimpanzees (15). Three of the *Pan troglodytes* chimpanzee subspecies are frequently infected in the wild with a lentivirus that ultimately was derived from simian immunodeficiency viruses infecting Old World monkey species (16). The hypothesis for an equivalent nonhuman primate origin for HCV fueled several published (17,18) and unpublished (S. Lyons et al., unpub. data) surveys for HCV or HCV-like viruses in chimpanzees, other apes, and a variety of Old World monkey species (19). These studies were encouraged by the serendipitous detection of a virus distantly related to HCV, termed GB virus B (GBV-B), in a laboratory-housed tamarin, a New World monkey (20,21). This detection was speculated to represent, in evolutionarily terms, the New World monkey homolog of HCV, a scenario that supports the possibility for the widespread distribution of further HCV-like viruses among Old World monkey species in Africa and Asia. Despite the plausibility of this hypothesis, we found that no survey to date has detected or obtained serologic evidence for infection with HCV or HCV-like viruses in any ape or Old World monkey species screened in published data (17,18) or in the aforementioned unpublished data of Lyons et al. As a further puzzling observation, GBV-B infection has not been reported in any other tamarin or other New World monkey species either in the wild or among captive animals. Like HCV, its origin remains unknown.

Considering this background and previous focus on primates for HCV origins, it came as a complete surprise that a virus, much more closely related to HCV than GBV-B, was recently described for domestic dogs (22). Its host and apparent tropism for the respiratory tract (and potential association with infectious respiratory disease) represent major differences from what might be expected for a close relative of HCV. It came as a further surprise that CHV RNA sequences were detected in plasma samples from 8 of 103 horses in New York state, USA (23). Results of a novel serologic test of samples from horses for antibodies to the conserved nonstructural protein 3 (NS3) showed an overall seropositivity of 35%, and 80 samples from dogs were negative by serologic testing and PCR. The wider host range of the virus implied by these findings led the authors to propose a new name, non-primate hepacivirus (NPHV) for CHV, and this new nomenclature is used in the current study.

To investigate the species distribution of NPHV or homologs in a range of mammalian species and to investigate clinical features of infection, we initiated large-scale PCR–based screening of plasma, respiratory, and postmortem liver, spleen, and lung samples from horses, dogs, cats, and other species originating in the United Kingdom. The PCR was specific for conserved sequences in the 5′ untranslated region (5′-UTR) and the NS3 regions of NPHV. NPHV was detected in 3 horses (horse 1, horse 2, and horse 3); initial characterization of the epidemiology and clinical features of NPHV infections were performed and compared with those of HCV.

## Materials and Methods

### Samples

A total of 552 samples were screened for the presence of NPHV RNA. Samples collected were from horses, dogs, cats, mice, pigs, and donkeys. All horse, cat, and donkey samples were sourced from either excess diagnostic samples or previously archived study samples from the Royal (Dick) School of Veterinary Studies, University of Edinburgh, where the laboratory investigation for this study was performed. Buccal swab samples were obtained from dogs (*Canis lupus familiaris*) undergoing veterinary examination at the Edinburgh Dog and Cat home. Bronchoalveolar lavage samples were collected from dogs undergoing investigation of respiratory disease.

Venus blood samples were collected from 353 nonprimates comprising 99 dogs, 158 horses and donkeys (142 *Equus ferus caballus, 16* *Equus africanus asinus*), 56 cats (*Felis catus*), 63 rodents (47 *Apodemus sylvaticus*, 8 *Mus muscullus*, 5 *Myodes glareolus*, 3 *Microtus agrestis*), and 40 pigs (*Sus scrofa*). Plasma was separated by centrifugation and frozen at −80°C until testing. Lung, liver, and spleen samples were obtained from dogs during autopsy at the pathology department of the school of veterinary studies and placed in RNAlater (QIAGEN, Crawley, UK) before RNA extraction. Samples of mouse liver were collected from all rodents in East Lothian, Scotland except 2 *Mus muscullus* for which liver samples were unavailable.

All clinical sampling was undertaken with full owner consent and in line with Royal (Dick) School of Veterinary Studies institutional and UK ethical guidelines.

### CHV and NPHV Screening

Screening for CHV and NPHV infections was performed by using PCR; serologic screening for antibodies against CHV/NPHV was precluded by the non-availability of NS3 antigen used in a previous study (23). To validate the PCR, RNA transcripts were generated from a plasmid containing partial CHV NS3 cDNA by using the Ambion T7 transcription kit (Promega Corp., Southampton, UK). Transcripts were purified with the RNeasy kit (QIAGEN), and concentrations were determined by using the NanoDrop 2000 (NanoDrop Products, Wilmington, DE, USA). RNA extractions were performed on 140 μL of plasma or respiratory sample by using the QIAmp viral extraction kit (QIAGEN) according to the manufacturer's instructions and eluted in a final volume of 60 μL. All tissue samples were homogenized in lysis buffer; RNA was extracted by using the RNeasy Mini Kit (QIAGEN) according to instructions and eluted in a final volume of 60 μL. Peripheral blood mononuclear cells were separated from whole blood immediately after collection by centrifugation on a Ficoll-Hypaque density gradient by using Histopaque 1077 according to manufacturer’s instructions (Sigma Aldrich, St. Louis, MO, USA), and RNA was extracted by using QIAmp RNA blood mini kit as instructed (QIAGEN) and eluted in final volume of 100 μL.

RNA was converted to cDNA by using random hexamers with the Reverse Transcription System A3500 (Promega) and then used in nested PCR with previously published CHV NS3 primers (22) Chv-0F1,Chv-0R1S1, Chv-0F2, and Chv-0R2 and new equine-based NS3 primers (Table 1) and amplified by using 2 rounds of 30 cycles at 94°C for 18 s, 50°C for 21 s, and 72°C for 1.5 min; and 1 cycle of 72°C for 5 min, with 2 μL of first-round amplicon added to the second round. Degenerate equine- and canine-based NS3 primers were designed on the basis of the sequence variability observed in the published NPHV sequences and used to additionally screen all samples from dogs and equids. The CHV NS3 transcript was tested by using both NS3 primer sets and used as a control in screening, with sensitivity of 0.5–5 RNA copies in a reaction (Tables 1,2).

**Table 1:** Transcript titration of nonprimate hepaciviruses with NS3 primers in samples from domestic horses, United Kingdom

Transcript RNA copies/mL	Published NS3	New NS3
5 x 10^6^	2/2	2/2
5 x 10^5^	2/2	2/2
5 x 10^4^	2/2	2/2
5,000	2/2	2/2
500	2/2	2/2
50	2/2	2/2
5	2/2	2/2
0.5	0/2	0/2
0	0/2	0/2

*NS, nonstructural protein.

**Table 2:** Nonprimate hepaciviruses primer sequences for 5′UTR, NS3, and NS5B in samples from horses, United Kingdom*

Primer	Position	Sequence, 5′ →3′
EQ5→UTROS	Forward outer sense	ACA YYA CCA TGT GTC ACT CCC CCT
EQ5→UTROAS	Reverse outer antisense	CYC ATG TCC TAT GGT CTA CGA GA
EQ5→UTRIS	Forward inner sense	ACA CGG AAA YGG GTT AAC CAY ACY C
EQ5→UTRIAS	Reverse inner antisense	GCC CTC GCA AGC ATC CTA TCA G
EQNS3OS	Forward outer sense	ATW TGT GAT GAR TGC CAY AGY AC
EQNS3OAS	Reverse outer antisense	TAG TAG GTB ACA GCR TTA GCY CC
EQNS3IS	Forward inner sense	TCY AAR GGT GTD AAG CTT GTT GT
EQNS3IAS	Reverse inner antisense	TGG CAG AAG YTA AGR TGY CTY CC
EQNS5BIS	Forward outer sense	AAR TGY TTT GAC TCY ACB GTC ACT C
EQNS5BOIAS	Reverse outer antisense	ACT RTG ACT RAT YGT YTC CCA ACT CG
EQNS5BIS2	Forward inner sense	CAY GAT ATA GAH ACT GAG AGR GA
EQNS5BIAS2	Reverse inner antisense	TCR TCT TCC TCR ACG CCY TTR CTG G

*UTR, untranslated region; NS, nonstructural protein.

To confirm positive results of screening, we designed degenerate primers derived from the NPHV sequences for the 5′-UTR and NS5B (Table 1). For all detected positive results, we used SuperScript III One-Step RT-PCR (Life Technologies, Paisley, UK) with 6 μL of RNA and cycling conditions as published (23) with 1 of the following first-round primer sets: EQ5→UTROS and EQ5→UTROAS or EQNS5BIS and EQNS5BIAS. From the first round, 2 uL was added to the second-round PCR with respective forward and reverse primer sets: EQ5→UTRIS and EQ5→UTRIAS or EQNSBIS2 and NS5BIAS2, with the following cycling conditions; 30 cycles at 94°C for 18 s, 50°C for 21 s, and 72°C for 1.5 min; and 1 cycle of 72°C for 5 min.

Positive second-round PCR amplicons were sequenced in both directions by using the inner sense and inner antisense primers used in the second round of amplification. Sequencing was executed by using Big Dye Terminator version 3.1 (Applied Biosystems, Paisley, UK) according to the manufacturer's instructions. Sequences were analyzed by using SSE version 1.0 software (24). Sequences obtained in this study have been assigned the GenBank accession nos. JX948116–JX948121.

Viral loads of positive samples were determined by real-time quantitative PCR and a standard calibration curve generated from a dilution series of the NS3 transcript. Dilutions were prepared from the CHV NS3 transcript from concentrations of 10^6^ to 1 copy/μL; 5 μL of transcript RNA was used to generate cDNA by using random hexamers and reverse transcription. Five-microliter aliquots of cDNA were assayed in triplicate for the 3 positive samples in the same way. To quantify viral loads of positive samples, EQNS3IS and EQNS3IAS primers were used with 4 μL of cDNA in the SensiFAST SYBR Hi-ROX Kit (BioLine, London, UK) per manufacturer’s instructions (Table 1) with the exception that the annealing temperature was reduced to 50°C and the extension time extended to 15 s. Samples were analyzed in triplicate and fluorescence measured by using the Rotor-Gene Q system (QIAGEN). Viral loads were read from the standard curve generated and converted to RNA copies/mL for sample volume used in extraction and elution of the RNA.

## Results

### Sample Screening

To investigate the frequency of NPHV infection in dogs, 46 respiratory samples collected from dogs over a 6-month period in the Edinburgh area were screened by published PCR-based screening methods (22,23) by using primers from the 5′-UTR and NS3 regions. An RNA transcript from the NS3 region (23) verified the sensitivity of the NS3-based assay to single RNA copies per amplification reaction (Table 2). All samples were negative in both genome regions (Table 3). Ninety-nine plasma samples from dogs that had a variety of clinical conditions and had been referred for virology screening, along with 15 autopsy lung, liver, and spleen samples from dogs, were additionally screened; results were uniformly negative in both regions.

**Table 3:** Nonprimate hepaciviruses sequences detected by using PCR on mammal samples, United Kingdom*

Animal/sample type	No.	NS3	5′-UTR	Published NS3	New primers, 5′-UTR
Dog					
Respiratory	53	0	0	0	0
Plasma	99	0	0	0	0
Lung	15	0	0	0	0
Liver	15	0	0	0	0
Spleen	15	0	0	0	0
Horse					
Respiratory	40	0	0	0	0
Plasma	142	3	3	3	3
Donkey					
Plasma	16	NA	NA	0	0
Cat					
Plasma	56	NA	NA	0	0
Pig					
Serum	40	NA	NA	0	0
Mouse					
Liver	61	NA	NA	0	0

*Data from *23.* NS, nonstructural protein; UTR, untranslated region; NA, not applicable.

Since publication of the NS3- and 5′-UTR–based PCRs (22), comparative sequence data from several NPHV-infected horses have become available (23). These data revealed sequence variability in the primer binding regions of both primer sets. We therefore redesigned the screening primers in both regions (Table 1) to accommodate this. In the 5′-UTR region, it was additionally possible to ensure that primers matched homologous regions of HCV genotypes 1–7. The new nested NS3 primers showed similar sensitivity for the NS3 transcript (Table 2). These new primers were used to repeat screening of all canine respiratory, plasma, and autopsy samples that produced uniformly negative results (Table 3).

To investigate the possible infection of non-canine mammalian species, we screened available plasma/serum, respiratory, and liver samples from horses (n = 175), donkeys (n = 16), domestic cats (n = 56), pigs (n = 40), and wild mice (n = 61) by using both sets of conserved primers (Table 3). From this extended survey, 3 plasma samples from horses were positive on initial screening and confirmed positive on reextraction and reamplification in 5′-UTR and NS3 regions. PCR of samples of all types from all other studied mammalian species showed negative results.

To confirm the presence of NPHV sequences in the 3 screen-positive horses, we further amplified each sample using conserved primers in the NS5B region and comparing amplified sequences from each region with homologous regions of previously identified positive horses (Figure). Although this method does not represent a comprehensive genetic analysis, these sequence comparisons demonstrated that each of the positive horses was infected with NPHV variants distinct from the transcript-positive control and from each of the 8 previously identified infected horses in the USA. All 3 variants showed similar branching orders in each genome region, consistent with the observed lack of recombination in previous analyses (23).

**Figure:**
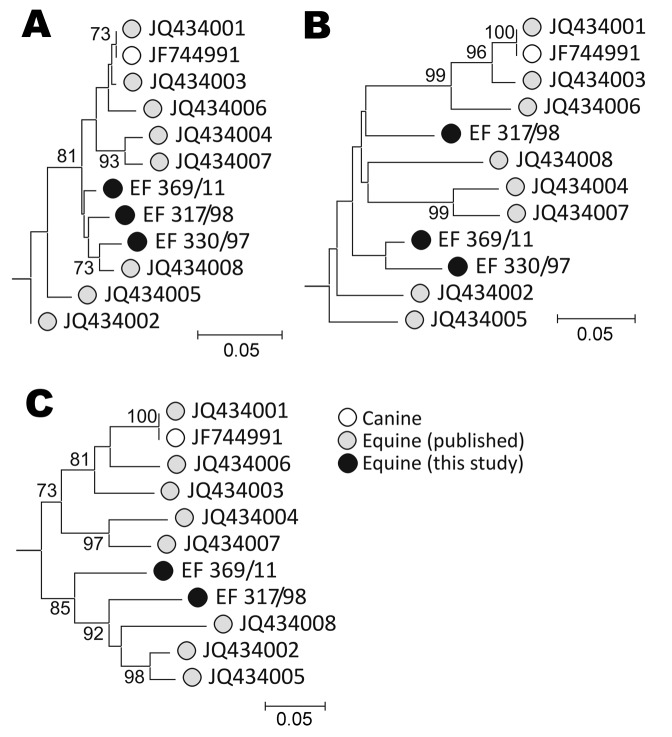
Phylogenetic analysis of A) 5′ untranslated region, B) nonstructural protein 3, and C) nonstructural protein 5B regions of nonprimate hepatitis virus sequence amplified from screen-positive study animals. Neighbor-joining trees of nucleotide sequences from each genome region were constructed from Jukes-Cantor corrected pairwise distances calculated by using the program MEGA version 5 (25; datasets were bootstrap re-sampled 500× to indicate robustness of branching [values >70% shown on branches]). The hepacivirus genotype 1a sequence, M62321, was used to root the tree (not shown). Scale bars indicate nucleotide substitutions per site.

### Virologic and Clinical Examination of NPHV-positive Horses

The 3 infected horses originated in Scotland and comprised 2 geldings and 1 mare, 12–20 years of age (Table 4). Clinical records of each horse from the time of sample collection failed to identify evidence of hepatitis or systemic disease. Liver function tests provided no evidence for hepatic inflammation: γ-glutamyl transferase (GGT) and glutamate dehydrogenase were within reference range, except a mildly elevated GGT level in horse 2 (Table 5). Testing also ruled out hepatic insufficiency: bile acid levels were within reference range. Viral loads, measured by using real-time PCR against the NS3 transcript standard, ranged from 7 × 10^4^ to 5 × 10^7^ RNA copies/mL among the 3 horses.

**Table 4:** Clinical features of domestic horses infected by nonprimate hepaciviruses, United Kingdom*

Horse	Sample	Collection date	Area	Age, y/sex	Presenting disease
1	EF_317/98	1998	Caithness	8/F	Lameness
2	EF_330/97	1997	Perthshire	12/M	Inflammatory airway disease
3	EF_369/11	2011 Dec 2	Lothian	20/M	Lameness; no lung disease
	EF_374/12	2012 Mar 1			Lameness; no lung disease
	EF_523/12	2012 Mar 23			Lameness; no lung disease

**Table 5:** Laboratory indices for domestic horses infected with nonprimate hepaciviruses, United Kingdom

Horse	Sample	GGT (<40 U/mL)	GLDH (<10 U/mL)	Bile acids (<10 U/mL)	Viral load, copies/mL
1	EF_317/98	15	2	1	1.3 × 10^5^
2	EF_330/97	59	2	3	4.4 × 10^5^
3	EF_369/11	15	2	6.4	4.8 × 10^7^
	EF_374/12	36	1	7.4	2.1 × 10^5^
	EF_523/12	24	4	6.3	7.1 × 10^4^

*GGT, γ-glutamyl transferase; GLDH, glutamate dehydrogenase.

To clinically characterize NPHV infection, we further examined 1 of the positive horses (horse 3) and collected samples at 4 and 5 months after the original sample collection. The horse remained clinically unremarkable and had no specific signs indicative of systemic disease. The horse regularly competed in equestrian events and had traveled extensively worldwide during the 10 years preceding detection of infection with NPHV. No specific risk factors, such as operations, exposure to unsterilized needles, or history of systemic illness were elicited by interviewing the owner. During the 5-month follow-up period, the horse remained viremic, but samples showed lower viral loads (7 × 10^4^ to2 × 10^5^ RNA copies/mL) than found in the initial sample (5 × 10^7^ copies/mL) (Table 5). Although liver indices were within the reference range, liver enzymes and bile acids were frequently at the upper end of the reference range. Although of questionable clinical significance, these observations and the elevated GGT level in horse 2 are potentially consistent with low-grade hepatitis. UK veterinary rules precluded taking a liver biopsy sample from horse 3 to further investigate this.

Nasal and mouth swab samples and peripheral blood mononuclear cells collected at month 5 were NPHV negative by PCR when both sets of primers were tested. Screening plasma samples from 6 horses stabled with horse 3 were uniformly NPHV negative in 5′-UTR and NS3 by PCR.

## Discussion

Although the study was designed as an investigation of the frequency of CHV infection in dogs, initial findings of uniformly negative results from a large number of respiratory, plasma, and autopsy samples prompted us to widen our sampling to other mammalian species. Consistent with a then-recent report (23), we found viruses similar to CHV and now termed NPHV in ≈3% of horse plasma samples but in no samples from other species (cats, pigs, or mice), irrespective of sample type (Table 3). The detection frequency in samples from horses 1, 2, and 3 was lower (but not significantly so by the Fisher exact test; p = 0.06) than the previous 8 from 103 viremia frequency among horses in New York state (23).

The restriction of NPHV infection to horses was consistent with serology-based screening (23) that showed 35% of samples from horses to contain antibodies against a recombinant NPHV NS3 peptide (of which ≈25% were additionally viremic) but an absence of NS3 antibodies in other species. These included dogs, deer and rabbits, although 1 intermediately seroreactive (but NPHV-negative by PCR) sample was found in 84 samples screened from cows. Whether these findings represent rare infection in another ruminant species or assay non-specificity requires further investigation. Although these initial surveys provide preliminary evidence that horses could be the natural host of NPHV, its previous detection in dogs with respiratory disease (22) provides evidence for its potential spread to humans. In this respect, NPHV specificity would differ from the narrow specificity observed in other hepaciviruses; e.g., HCV can infect humans and chimpanzees (although it does not naturally circulate in the latter species) and cannot infect Old World monkey species, such as macaques (26). The host range of GBV-B appears similarly restricted to New World monkeys (27). Further studies are needed to determine whether additional equally or more divergent hepaciviruses are distributed in other mammalian species; the 5′-UTR primers developed for the current study used with primers selective for regions conserved between NPHV and HCV might provide a useful assay for this purpose.

The detection of NPHV RNA sequences in samples obtained 5 months apart from horse 3 provides evidence for an ability of NPHV to establish persistent infections. Although longer term sampling is required to confirm this possibility, the observation is consistent with the high viremia frequencies among seropositive horses in a previous study (8/37) (23). This proportion would probably not be observed on random sampling if infections had rapidly resolved. In this respect, NPHV may at least partly reproduce the documented high rates of HCV persistence, in which >50% of those exposed showed decades- or life-long viremia and active liver disease in the absence of treatment. GBV-B, although clearly hepatotropic, does not establish persistent infections in tamarins or owl monkeys (20,27,28). However, more recent studies have demonstrated long-term persistence among experimentally infected marmosets (29,30).

Neither the clinical signs nor the liver function tests of the 3 NPHV-infected horses provided a clear indication of the organism’s association with hepatic or other systemic disease (Tables 4, 5). GGT and glutamate dehydrogenase are sensitive markers of liver inflammatory processes in the horse but with 1 exception were in the reference range. Reference levels of bile acids similarly demonstrated adequate liver function. Although the sample size was small, these relatively normal liver indices contrast with the frequent GGT and ALT elevations associated with chronic HCV infection and found in New World monkeys experimentally infected with GBV-B. Although in the current study, UK veterinary regulations did not permit liver biopsies to be performed on horses without evidence of liver disease, the current findings do not rule out a lower grade infection or potential replication in the liver without the associated immunologic response to HCV that is primarily responsible for liver damage (31,32). Future studies, perhaps refocused on NPHV screening of horses with idiopathic liver disease that have undergone biopsy sampling and have been clinically characterized, are needed to investigate further the potential for hepatotropic NPHV and manifest its clinical effects. In the longer term, and acknowledging that the horse is not the ideal experimental animal, inoculation of horses with NPHV and subsequent monitoring for viremia development, liver function abnormalities, and B- and T-cell immune responses would provide further insights into the nature of NPHV infections and associated immune response and similarity of these developments to current observations for HCV and GBV-B.
